# Rewiring the Senses: The Impact of Sensory Integration Therapy on Balance and Cognition in Cerebral Palsy

**DOI:** 10.1155/oti/3715445

**Published:** 2026-06-10

**Authors:** Hande Yılmaz, Feyza Şule Badıllı Hantal

**Affiliations:** ^1^ Department of Physiotherapy and Rehabilitation, Yeditepe University, Istanbul, Türkiye, yeditepe.edu.tr

**Keywords:** activities of daily living, cerebral palsy, cognition, postural balance, sensory integration

## Abstract

**Aim:**

The aim of this study was to investigate the effects of sensory integration therapy on balance, functional independence, functional mobility, sensory processing, and cognitive function in children with cerebral palsy.

**Methods:**

Twenty‐two children with cerebral palsy attending a rehabilitation center participated in the study. Participants were randomly assigned to the study group (two females, nine males; 7.3 ± 1.7 years) and the control group (four females, seven males; 8.3 ± 1.4 years). Sociodemographic data were collected using a structured questionnaire. Balance was assessed with the **Pediatric Berg Balance Scale**, functional mobility with the **Timed Up and Go Test**, functional independence with **WeeFIM**, sensory processing with the **Sensory Profile**, and cognitive function with Dynamic Occupational Therapy Cognitive Assessment for Children (DOTCA‐Ch). Both groups received balance and coordination exercises (1 session/week, 45 minutes), while the study group additionally received individualized sensory integration therapy (1 session/week, 45 min) for 12 weeks.

**Results:**

Both groups showed improvement in all measured variables after the intervention. Although both groups demonstrated improvements in Berg Balance Scale and Timed Up and Go Test scores following the intervention, no statistically significant between‐group differences were observed (*p* > 0.05). Significant differences (*p* < 0.05) were found between groups in **WeeFIM**, **DOTCA-Ch**, and specific subscales of the **Sensory Profile** (sensory seeking, emotional response, inattention, sensory sensitivity, and perceptual fine motor domains).

**Conclusion:**

Consistent with the hypothesis, sensory integration therapy combined with balance and coordination exercises was associated with improvements in balance, functional mobility, independence, sensory processing, and cognitive function in children with cerebral palsy. However, these findings should be interpreted cautiously because formal group × time interaction analyses were not performed.

## 1. Introduction

Cerebral palsy (CP) encompasses a group of permanent disorders of movement and posture caused by disturbances in the developing fetal or infant brain. Although the brain lesion itself is nonprogressive, its clinical manifestations may change over time as the child grows and develops [1]. In addition to motor dysfunction, children with CP often present with sensory, perceptual, cognitive, behavioral, and communication problems. Epileptic seizures and secondary musculoskeletal complications are also common. Motor limitations frequently necessitate lifelong individualized rehabilitation. Alongside impaired voluntary muscle control and coordination, 50%–75% of children with CP exhibit intellectual disability or learning difficulties, 25%–35% experience epileptic seizures, approximately 25% have speech or hearing disorders, and 40%–50% present with visual impairments [2].

In all clinical types of CP, motor deficits are regarded as the primary problem; however, these are often accompanied by sensory and cognitive disturbances. The underlying causes of motor dysfunction include abnormal muscle tone, weakness, and involuntary movements of the limbs and trunk. Although the brain lesion responsible for CP is nonprogressive, the clinical presentation can evolve as the child grows. Central nervous system injury adversely affects posture and movement, leading to limitations in balance reactions, motor development, and functional independence [3, 4].

Sensory modulation refers to the ability to perceive sensory input and generate adaptive responses appropriate to situational demands. It involves maintaining an optimal level of arousal, organizing sensory input, filtering out irrelevant stimuli, and attending to relevant ones. This process is fundamental for effective interaction with the environment, adaptation to daily challenges, and overall quality of life [5]. Sensory modulation disorders are characterized by atypical responses to sensory input, reflecting difficulties in the regulation, organization, and adaptive use of sensory information across multiple modalities. Such disturbances may manifest as sensory overresponsivity, underresponsivity, or sensory seeking behaviors and are associated with challenges in attention, emotional regulation, and motor planning, ultimately affecting participation in daily activities and functional independence [6, 7].

Many children with CP display not only neuromotor impairments but also deficits in sensory processing and praxis. Perceptual, cognitive, communication, and behavioral difficulties may further limit their activity levels and participation in daily life. Sensory integration is a dynamic process by which the nervous system synthesizes and organizes sensory input from the body and environment to generate goal‐directed, purposeful responses. Proper sensory integration facilitates the development of body schema, postural stability, motor planning, bilateral coordination, and hand–eye coordination [8, 9].

Sensory integration therapy (SIT) is a therapeutic approach developed to evaluate and address deficits in sensory processing and perceptual–motor performance in children with neurological and developmental disorders, including cerebral palsy. Because of their limited motor abilities, these children often have reduced opportunities for rich sensory experiences, which may further restrict neuroplastic adaptation and developmental progression. SIT provides structured, multisensory input—encompassing tactile, proprioceptive, vestibular, visual, and auditory modalities—through active, play‐based motor engagement. This controlled sensory stimulation aims to facilitate adaptive responses, enhance sensory–motor integration, and ultimately improve postural control, motor planning, and functional performance [10, 11].

Recent clinical and experimental evidence has highlighted the therapeutic potential of sensory integration‐based interventions in children with neurodevelopmental disorders. In particular, studies involving children with cerebral palsy have demonstrated that incorporating SIT into conventional neurorehabilitation programs can lead to measurable improvements in spasticity, balance, postural stability, and gross motor function. These benefits are attributed to enhanced multisensory processing, improved sensory–motor coupling, and increased cortical plasticity following structured sensory–motor stimulation [12, 13]. In a recent controlled intervention study involving children with spastic diplegic cerebral palsy, the incorporation of SIT into conventional physiotherapy protocols produced significantly greater improvements in postural balance and gross motor performance—measured by the Pediatric Balance Scale and Gross Motor Function Measure—compared with conventional therapy alone, supporting the additive role of multisensory stimulation in motor rehabilitation [14]. Moreover, research in related populations, such as autism spectrum disorder, indicates that sensory integration training can enhance balance and executive function performance, supported by neuroimaging and biomechanical evidence [15]. These findings suggest that SIT may exert effects beyond motor control—potentially affecting sensory processing and higher order cognitive domains—yet evidence in children with CP remains scarce and inconsistent.

Emerging evidence suggests that SIT may influence not only motor performance but also higher order cognitive processes. Effective sensory processing is closely associated with attention, motor planning, executive functioning, and adaptive behavioral responses, all of which contribute to learning and participation in daily activities. Structured multisensory experiences provided during SIT are thought to facilitate neuroplastic adaptation and improve sensory–motor coupling, which may indirectly support cognitive performance in children with cerebral palsy [12]. Furthermore, studies in related neurodevelopmental populations have reported improvements in executive function and balance following sensory integration‐based interventions [15]. However, evidence regarding the cognitive effects of SIT in this population remains limited. Therefore, the present study aimed to investigate the effects of SIT on both motor‐related outcomes and cognitive function in children with cerebral palsy.

Despite growing interest in sensory‐based interventions, limited research has explored the effects of SIT on balance and cognitive function in children with cerebral palsy. The present study hypothesizes that combining balance–coordination exercises with SIT will enhance balance and cognitive performance in children with CP aged 6–10 years. Therefore, this study aims to investigate the effects of SIT on balance, functional independence, functional mobility, sensory processing, and cognitive function in children with cerebral palsy.

## 2. Materials and Methods

### 2.1. Participants and Study Design

A total of 28 children diagnosed with different types of cerebral palsy (CP), according to their medical records and the study′s eligibility criteria, were recruited from *Altın Adımlar Psychological Counseling and Active Life Center*, a private special education and rehabilitation facility. This study was approved by the *Bahçeşehir University Clinical Research Ethics Committee* (approval date: October 31, 2017). A priori power analysis was performed based on the expected changes in DOTCA‐Ch outcomes. A priori power analysis was performed using G∗Power software (version 3.1; [16]). Assuming a large effect size (Cohen′s *d* = 0.80), an alpha level of 0.05, and 80% statistical power, the minimum required sample size was calculated as 10 participants per group. Written informed consent was obtained from the parents or legal guardians of all participants, in accordance with institutional ethical standards.

**Figure 1:**
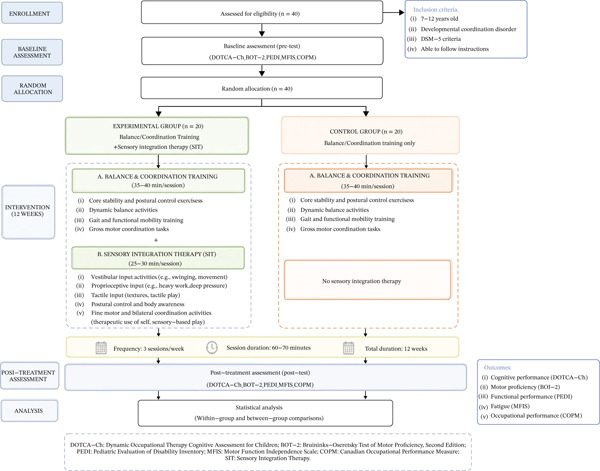
Schematic flowchart of the study design and intervention protocol.

Participants were assigned using a simple random allocation procedure based on a computer‐generated random sequence. The experimental group received SIT combined with balance and coordination exercises, whereas the control group received only balance and coordination exercises. Both programs were conducted for 12 consecutive weeks, with one session per week, each lasting 45 min. All sessions were administered by the same pediatric physiotherapist trained in neurodevelopmental treatment, ensuring standardization and consistency across sessions. A schematic flowchart summarizing participant allocation, intervention procedures, and assessment timeline is presented in Figure 1.

Eligible participants were children aged 6–10 years, diagnosed with cerebral palsy, classified at Gross Motor Function Classification System (GMFCS) Levels I or II, able to comprehend and follow verbal instructions, and without sensory deficits (hearing or vision) that could interfere with communication. Children were excluded if they had received Botulinum toxin‐A (BTX‐A) treatment in the previous 6 months, had undergone orthopedic surgery of the hip, knee, or ankle in the past year, presented with severe intellectual disability preventing participation, or did not consent to join the study.

### 2.2. Assessments

All participants were assessed before (pretreatment, T₀) and after the 12‐week intervention (posttreatment, T₁) using standardized and validated outcome measures. All evaluations were performed by the same physiotherapist, who was blinded to group assignment to reduce measurement bias.

A structured Patient Information Form was developed to collect demographic and clinical data. The form included details on age, sex, height, weight, educational status, presence of chronic diseases, and medications used. Information regarding birth and family history, previous surgeries, botulinum toxin applications, orthosis use, and participation in earlier physiotherapy or rehabilitation programs (start age, duration, and frequency) was also obtained.

Additionally, participants′ motor developmental milestones (e.g., head control, sitting, crawling, and walking) were documented. A detailed history of falls, including frequency, cause, circumstances, location, and injury outcomes, was recorded to support functional and balance assessments.

### 2.3. Assessment Procedures

To ensure standardized data collection and minimize measurement bias, all outcome assessments were performed under consistent testing conditions before and after the intervention period. The assessments were selected to evaluate balance, functional mobility, functional independence, sensory processing, and cognitive function in children with cerebral palsy.

All assessments were conducted individually in a quiet and standardized rehabilitation room by the same pediatric physiotherapist, who was experienced in pediatric neurorehabilitation and blinded to group allocation. Prior to testing, standardized verbal instructions and demonstrations were provided to each participant to ensure task comprehension. Participants were allowed sufficient rest periods between assessments to minimize fatigue.

The Pediatric Berg Balance Scale (PBS), Timed Up and Go (TUG) Test, Sensory Profile, Functional Independence Measure for Children (WeeFIM), and Dynamic Occupational Therapy Cognitive Assessment for Children (DOTCA‐Ch) were administered according to standardized assessment procedures as described below:

### 2.4. PBS

The PBS is an adaptation of the original Berg Balance Scale (BBS), designed to assess functional balance abilities in children during everyday activities. The scale evaluates a child′s capacity to maintain static and dynamic postural control while voluntarily shifting the body′s center of gravity over a progressively reduced base of support [17–19].

The PBS consists of 14 items, each scored on a 5‐point ordinal scale (0–4), with higher scores indicating better balance performance. The maximum total score is 56 points, representing excellent balance ability, while lower scores correspond to increased risk of falls. Specifically, scores between 41 and 56 suggest independent ambulation with minimal fall risk, 21 and 40 indicate moderate risk requiring support for ambulation, and 0 and 20 reflect a high likelihood of wheelchair dependence [18].

In the pediatric version, the sequence of test items was reorganized from simple to complex to better match developmental stages, and time standards for maintaining postural positions were adjusted to suit children′s capacities. Additionally, the test instructions were modified to ensure age‐appropriate comprehension and engagement during testing [18, 19].

The PBS has been shown to have excellent reliability (ICC > 0.98) and internal consistency (Cronbach′s *α* = 0.99) in children with cerebral palsy, and it is widely used in pediatric neurorehabilitation research for evaluating functional balance outcomes [18].

### 2.5. TUG Test

The TUG test is a widely used clinical measure for assessing functional mobility, integrating components of strength, dynamic balance, and agility. It is applicable across various populations, including children, adults, and older individuals, and provides a simple yet reliable indicator of postural control and movement efficiency [20, 21].

During the test, the participant is instructed to stand up from a seated position, walk 3 m, turn around, return to the chair, and sit down. The total time required to complete the task is recorded in seconds using a stopwatch. Shorter completion times indicate better functional mobility and lower fall risk. In general, a score of 10 s or less reflects independent mobility with minimal fall risk, whereas scores above 30 s suggest limited mobility and increased risk of falls [21, 22].

In this study, the TUG test was performed three times, and the mean value of the three trials was used for analysis to enhance reliability and reduce measurement error.

### 2.6. Sensory Profile

The Sensory Profile is a standardized, caregiver‐completed questionnaire designed to evaluate sensory processing patterns and their effects on functional performance in children aged **3 to** 10 years. The instrument consists of 125 items, each representing behaviors that reflect responses to everyday sensory experiences (1,2). Caregivers rate the frequency of each behavior using a 5‐point Likert scale ranging from 1 (*Always*) to 5 (*Never*).

The test includes 14 sections that assess three major domains: sensory processing, modulation, and behavioral and emotional responses to sensory input. After caregivers are instructed on how to complete the form, they are asked to select the options that best describe their child′s typical behaviors. Raw scores for each section are compared to norm‐referenced cutoff values, which categorize performance into interpretive ranges (e.g., typical, probable difference, and definite difference). A lower raw score indicates greater sensory processing difficulty [23].

The Sensory Profile is based on Dunn′s Model of Sensory Processing, which classifies sensory responses into four quadrants: sensory sensitivity, sensation avoiding, low registration, and sensation seeking (1). The questionnaire typically requires approximately 30 min to complete and an additional 20–30 min for scoring and interpretation by the clinician [23].

The Sensory Profile has been widely validated and demonstrates excellent internal consistency (Cronbach′s *α* = 0.89–0.95) and test–retest reliability (*r* = 0.80–0.90) across diverse pediatric populations, including children with cerebral palsy [24, 25].

### 2.7. Functional Independence Measure for Children (WeeFIM)

The Functional Independence Measure for Children (WeeFIM) is an adaptation of the adult Functional Independence Measure, developed to assess functional independence and developmental performance in children with cerebral palsy and other developmental disorders. It evaluates self‐care, mobility, and cognitive–social abilities, providing a comprehensive measure of assistance required in daily activities. The scale consists of 18 items across six domains—self‐care, sphincter control, transfers, locomotion, communication, and social cognition—each scored from 1 (*Total assistance*) to 7 (*Complete independence*). Total scores range from 18 to 126, with higher scores reflecting greater independence. The WeeFIM has demonstrated strong reliability (ICC > 0.95) and validity in children aged 6 months to 12 years and is widely used in pediatric neurorehabilitation for monitoring functional outcomes [26, 27].

### 2.8. DOTCA‐Ch

**Figure 2:**
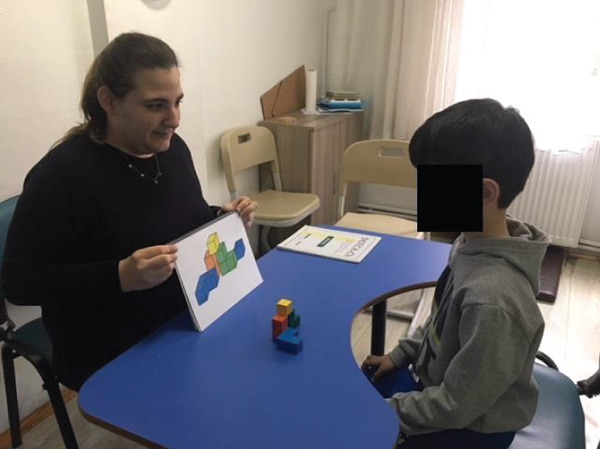
Example of cognitive assessment using the Dynamic Occupational Therapy Cognitive Assessment for Children (DOTCA‐Ch) during the intervention process.

The DOTCA‐Ch is a standardized tool developed to directly evaluate cognitive performance and learning potential in children aged 6 to 12 years. It is based on a dynamic assessment approach, which examines not only current cognitive abilities but also a child′s capacity to benefit from guided learning and mediation [28]. The DOTCA‐Ch is designed to identify the cognitive strengths, limitations, and learning strategies of children with developmental, cognitive, or learning difficulties, as well as those with acquired brain injury. It enables clinicians to determine the need for occupational therapy intervention and to plan individualized cognitive rehabilitation programs (Figure 2). The assessment comprises five cognitive domains—orientation, spatial perception, praxis, visuomotor construction, and thinking operations—consisting of 22 subtests. Immediate and delayed memory components are embedded within the visuomotor construction domain. Administration time is recorded for selected subtests to assess processing efficiency and planning ability [28, 29]. The present study utilized the standardized DOTCA‐Ch test (Serial No. 71823‐0000).

### 2.9. Treatment Protocol

Both the experimental and control groups received a standard physiotherapy program focusing on individualized static and dynamic balance and coordination exercises, tailored to each child′s specific needs. The intervention was delivered once per week for 12 weeks, with each session lasting 45 min and administered by the same pediatric physiotherapist. The intervention frequency and duration were determined considering participant adherence, school schedules, clinical feasibility, and continuity of rehabilitation. A structured once‐weekly, 45‐min intervention program over 12 weeks was considered feasible for maintaining regular participation throughout the study period, consistent with previous sensory integration‐based rehabilitation research in pediatric neurological populations. The core exercises included tandem standing and walking, side‐stepping, standing with eyes open and closed, single‐leg stance, and Frenkel coordination exercises, all designed to enhance postural control and balance stability.

**Figure 3:**
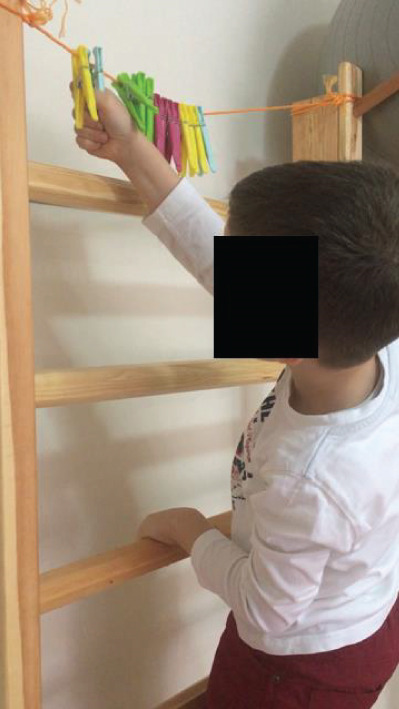
Example of a sensory integration therapy‐based upper‐extremity coordination and fine motor activity performed during the intervention program.

In addition to the standard program, the experimental group received SIT emphasizing vestibular, tactile, and proprioceptive stimulation, with a focus on praxis‐oriented activities. During SIT sessions, therapy materials and environmental stimuli were selected based on each child′s motivation, physical ability, and sensory responsiveness. The therapeutic setting was arranged to be stimulating, supportive, and motivating, whereas both verbal and visual cues were used to sustain attention and engagement (Figure 3).

The SIT program was based on sensory integration principles emphasizing active participation, adaptive responses, and multisensory stimulation. The intervention primarily targeted vestibular, proprioceptive, tactile, visual, and auditory sensory processing through play‐based and task‐oriented activities individualized according to each child′s sensory and motor characteristics. SIT sessions included activities designed to improve postural control, bilateral coordination, motor planning, body awareness, and praxis skills. Therapeutic activities were progressively modified according to the child′s functional performance and sensory responsiveness throughout the intervention period. The theoretical framework of the intervention was based on sensory integration approaches commonly applied in pediatric neurorehabilitation [11, 13].

To ensure optimal participation, potential stressors that could negatively affect the child′s motivation were minimized. All therapy sessions were individualized, taking into account the child′s motor severity, cognitive level, sensory processing abilities, attention span, and cooperation throughout the intervention process. Exercise selection was individualized according to each child′s motor function level, balance performance, sensory processing characteristics, cooperation, and tolerance to activity. The intervention protocol followed a progressive structure in which task complexity and postural challenge were gradually increased based on the child′s functional performance throughout the 12‐week period.

Each treatment session lasted 45 min and included balance, coordination, and sensory integration‐based activities performed in repeated task‐oriented practice periods with rest intervals as needed to prevent fatigue. Activities were adapted and progressed by modifying base of support, movement complexity, sensory input, and dual‐task demands according to participant performance and therapeutic response.

### 2.10. Statistical Analysis

All data analyses were performed using the Statistical Package for the Social Sciences (SPSS) version 22.0 (IBM Corp., Armonk, NY, USA). A *p* value < 0.05 was considered statistically significant. Descriptive statistics, including mean, standard deviation, median, minimum, maximum, frequency, and percentage values, were calculated for all variables. The Kolmogorov–Smirnov test was applied to assess the normality of data distribution. Because of the relatively small sample size and nonnormal distribution of several variables, nonparametric statistical methods were preferred. Mann–Whitney *U* tests were used for between‐group comparisons, whereas Wilcoxon signed‐rank tests were applied for within‐group analyses. Although this analytical approach does not directly evaluate group × time interaction effects and may limit causal interpretation of intervention‐related differences, it was considered appropriate given the relatively small sample size and the nonnormal distribution of several variables. For independent quantitative variables, the Mann–Whitney *U* test was used; for dependent quantitative variables, the Wilcoxon signed‐rank test was applied. For categorical data, the chi‐square test was used, and when the test assumptions were not met, the Fisher′s exact test was employed.

## 3. Results

Of the 28 participants initially enrolled in the study, six were excluded from the final analysis: three discontinued participation in the rehabilitation program, two withdrew for personal reasons, and one underwent surgery during the study period, which resulted in exclusion according to the predefined criteria. Therefore, the final analyses were conducted with 22 participants who completed the intervention protocol.

The demographic and physical characteristics of the experimental and control groups are presented in Table 1. No statistically significant differences were observed between the groups regarding age or sex distribution (*p* > 0.05). However, significant between‐group differences were identified for height, body weight, and body mass index (BMI), with the control group demonstrating higher values compared with the experimental group (*p* < 0.05).

**Table 1:** Demographic and physical characteristics of the participants.

Variable	Experimental group (*m* *e* *a* *n* ± *S* *D*/*n*–*%*)	Control group (*m* *e* *a* *n* ± *S* *D*/*n*–*%*)	*Z*/*χ* ^2^	*p*
Age (years)	7.3 ± 1.7	8.3 ± 1.4	−1.428ᵐ	0.153
Sex			0.917ˣ	0.338
Female	2 (18%)	4 (36%)		
Male	9 (82%)	7 (64%)		
Height (cm)	119.7 ± 7.3	125.8 ± 6.9	−2.276ᵐ	0.023
Weight (kg)	24.7 ± 6.4	33.7 ± 6.5	−3.059ᵐ	0.002
BMI (kg/m^2^)	17.1 ± 2.9	21.1 ± 2.4	−2.857ᵐ	0.004

*Note:* ᵐ Mann–Whitney *U* test ˣ Chi‐square test.

Analysis of the Sensory Profile subscales revealed no significant baseline differences between the experimental and control groups in the Registration, Sensitivity, and Avoiding domains, whereas a significant difference was observed in the Seeking subscale (Table 2). Posttreatment between‐group comparisons did not demonstrate statistically significant differences across sensory processing subdomains (Table 2).

**Table 2:** Comparison of pre‐ and posttreatment scores for sensory processing patterns of sensory processing between the study and control groups.

			Experimental group (mean ± SD)	Control group (mean±SD)	*Z*	*p*	Test
Sensory profile (sensory processing patterns)	Registration	Pretreatment	55.9 ± 7.1	59.9 ± 10.8	−1.252	0.211	m
		Posttreatment	60.9 ± 7.0	62.3 ± 11.0	−0.329	0.742	m
		Pre‐/postchange	5.0 ± 3.7	2.4 ± 1.0	−1.672	0.095	m
		Pre‐/postphange *p*	0.003	0.003			w
	Seeking						
		Pretreatment	101.5 ± 18.0	120.9 ± 9.4	−2.826	0.005	m
		Posttreatment	107.5 ± 14.2	121.7 ± 8.3	−2.499	0.012	m
		Pre‐/Post Change	6.1 ± 5.4	0.8 ± 1.3	−2.938	0.003	m
		Pre‐/postchange *p*	0.007	0.066			w
	Sensitivity						
		Pretreatment	78.9 ± 12.0	86.3 ± 6.2	−0.924	0.355	m
		Posttreatment	83.5 ± 10.4	87.5 ± 6.7	−0.594	0.553	m
		Pre/Post Change	4.6 ± 3.6	1.3 ± 1.3	−2.367	0.018	m
		Pre‐/postchange *p*	0.005	0.025			w
	Avoiding		Experimental group (*m* *e* *a* *n* ± *S* *D*)	Control group (*m* *e* *a* *n* ± *S* *D*)	*Z*	*p*	Test
		Pretreatment	117.6 ± 11.3	125.5 ± 14.7	−1.544	0.122	m
		Posttreatment	124.7 ± 11.1	127.0 ± 14.1	−0.592	0.554	m
		Pre‐/postchange	7.1 ± 5.2	1.5 ± 1.2	−2.748	0.006	m
		Pre‐/postchange *p*	0.005	0.007			w

*Note:* Values are presented as mean ± standard deviation. Within‐group changes were analyzed using the Wilcoxon signed‐rank test, and between‐group comparisons were performed using the Mann–Whitney U test. A p value < 0.05 was considered statistically significant.

Evaluation of pre‐ to posttreatment changes showed larger change scores in the experimental group for the Seeking, Sensitivity, and Avoiding subscales, whereas the magnitude of change in Registration did not significantly differ between groups (Table 2).

Within‐group analyses demonstrated significant pre‐ to posttreatment improvements across all sensory processing subscales in the experimental group. In the control group, significant within‐group changes were observed in Registration, Sensitivity, and Avoiding, whereas no significant change was identified in the Seeking subscale (Table 2).

Behavioral and motor components of sensory processing are presented in Table 3. At baseline, no statistically significant differences were observed between the experimental and control groups across most subdomains; however, significant between‐group differences were identified for oral sensitivity and sensory input seeking.

**Table 3:** Comparison of pre‐ and posttreatment scores for behavioral and motor components of sensory processing between the study and control groups.

			Study group (mean ± SD)	Control group (mean ± SD)	*Z*	*p*	m
Sensory processing (behavioral and motor components)	Low endurance	Pretreatment	34.0 ± 5.5	33.3 ± 6.1	−0.264	0.792	m
		Posttreatment	37.3 ± 5.3	33.4 ± 6.7	−0.659	0.510	m
		Pre‐/postchange	3.3 ± 2.9	1.5 ± 1.0	−1.650	0.099	m
		*p* for pre‐/postchange	0.003	0.007			w
	Oral sensitivity		Study group (*m* *e* *a* *n* ± *S* *D*)	Control group (*m* *e* *a* *n* ± *S* *D*)	*Z*	*p*	m
		Pretreatment	33.2 ± 8.2	43.5 ± 2.3	−3.368	0.001	m
		Posttreatment	35.5 ± 6.7	43.5 ± 2.2	−3.140	0.002	m
		Pre‐/postchange	2.3 ± 2.5	0.1 ± 1.3	−3.067	0.002	m
		*p* for pre‐/postchange	0.011	0.317			w
	Inattention		Study group (*m* *e* *a* *n* ± *S* *D*)	Control group (*m* *e* *a* *n* ± *S* *D*)	*Z*	*p*	
		Pretreatment	26.3 ± 6.3	29.3 ± 2.9	−0.893	0.372	m
		Posttreatment	28.5 ± 4.9	30.0 ± 2.7	−0.594	0.553	m
		Pre‐/postchange	2.2 ± 1.9	0.7 ± 0.6	−2.031	0.042	m
		*p* for pre‐/postchange	0.007	0.011			w
	Weak Registration		Study group (*m* *e* *a* *n* ± *S* *D*)	Control group (*m* *e* *a* *n* ± *S* *D*)	*Z*	*p*	
		Pretreatment	36.2 ± 3.2	38.3 ± 3.0	−1.912	0.052	m
		Posttreatment	36.5 ± 3.0	38.3 ± 3.0	−1.789	0.074	m
		Pre‐/postchange	0.4 ± 0.7	0.0 ± 0.0	−1.817	0.069	m
		*p* for pre‐/postchange	0.102	1.000			w
	Sensory sensitivity		Study group (*m* *e* *a* *n* ± *S* *D*)	Control group (*M* *e* *a* *n* ± *S* *D*)	*Z*	*p*	
		Pretreatment	14.1 ± 3.6	15.5 ± 3.7	−0.660	0.509	m
		Posttreatment	15.5 ± 3.4	16.1 ± 3.5	−0.168	0.869	m
		Pre‐/postchange	1.5 ± 1.4	0.5 ± 0.8	−1.590	0.112	m
		*p* for pre‐/postchange	0.017	0.063			w
	Sedentary		Study group (*m* *e* *a* *n* ± *S* *D*)	Control group (*m* *e* *a* *n* ± *S* *D*)	*Z*	*p*	
		Pretreatment	15.5 ± 4.8	14.6 ± 4.1	−0.603	0.547	m
		Posttreatment	16.2 ± 4.1	14.9 ± 3.8	−0.837	0.403	m
		Pre/postchange	0.6 ± 1.1	0.3 ± 1.3	−0.672	0.502	m
		*p* for pre‐/postchange	0.102	0.180			w
	Perceptual fine motor		Study group (*m* *e* *a* *n* ± *S* *D*)	Control group (*m* *e* *a* *n* ± *S* *D*)	*Z*	*p*	
		Pretreatment	8.8 ± 3.5	11.0 ± 1.8	−1.662	0.097	m
		Posttreatment	10.4 ± 3.1	11.5 ± 1.9	−1.292	0.196	m
		Pre‐/postchange	1.5 ± 1.2	0.5 ± 0.5	−2.185	0.029	m
		*p* for pre‐/postchange	0.011	0.025			w

*Note:* Values are presented as mean ± standard deviation. Within‐group changes were analyzed using the Wilcoxon signed‐rank test, and between‐group comparisons were performed using the Mann–Whitney U test. A p value < 0.05 was considered statistically significant.

Posttreatment between‐group comparisons did not demonstrate statistically significant differences in absolute scores across subdomains. Analysis of pre‐ to posttreatment change scores demonstrated significant between‐group differences in oral sensitivity, inattention, perceptual fine motor skills, sensory input seeking, and emotional response, with larger changes observed in the experimental group (Table 3). No significant between‐group differences in change scores were identified for low endurance, weak registration, sensory sensitivity, or sedentary behavior.

Within‐group analyses demonstrated significant pre‐ to posttreatment changes in several subdomains in the experimental group, whereas within‐group changes in the control group were more limited and not consistently significant across all subdomains.

Changes in sensory processing patterns are summarized in Table 2. In addition to these general sensory domains, behavioral and motor components of sensory processing were further evaluated and are presented in Table 3.

Analysis of Table 3 demonstrated that posttreatment scores were generally comparable between groups. However, significant between‐group differences in pre‐ to posttreatment change scores were identified for oral sensitivity, inattention, perceptual fine motor skills, sensory input seeking, and emotional response, with larger changes observed in the experimental group (Table 3). No significant between‐group differences in change scores were observed for low endurance, weak registration, sensory sensitivity, or sedentary behavior.

Cognitive performance assessed by the DOTCA‐Ch is presented in Table 4. At baseline, no statistically significant differences were observed between the experimental and control groups across cognitive subdomains.

**Table 4:** Pre‐ and posttreatment comparison of DOTCA‐CH cognitive domain scores between the study and control groups.

			Study group mean±SD	Control group mean±SD	*Z*	*p*	Test
DOTCA‐CH	Orientation	Pretreatment	10.4 ± 3.1	11.3 ± 4.2	−0.498	0.618	m
		Posttreatment	12.2 ± 2.8	11.4 ± 4.2	−0.433	0.665	m
		Pre‐/postdifference	1.8 ± 1.3	0.1 ± 1.5	−3.155	0.002	m
		Within‐group *p*	0.010	0.317			w
	Spatial perception						
		Pretreatment	9.2 ± 1.5	8.0 ± 2.2	−1.321	0.187	m
		Posttreatment	10.7 ± 1.6	8.2 ± 2.4	−2.425	0.015	m
		Pre‐/post‐difference	1.5 ± 1.1	0.2 ± 0.6	−2.911	0.004	m
		Within‐group *p*	0.010	0.317			w
	Praxis						
		Pretreatment	21.1 ± 8.5	22.5 ± 9.4	0.000	1.000	m
		Posttreatment	29.1 ± 6.8	24.5 ± 9.6	−1.350	0.177	m
		Pre‐/postchange	8.0 ± 3.5	2.1 ± 4.5	−4.015	0.000	m
		Pre‐/postchange *p*	0.003	0.002			w
	Visuomotor construction						
		Pretreatment	22.5 ± 5.5	22.0 ± 6.1	−0.329	0.742	m
		Posttreatment	25.5 ± 5.5	22.9 ± 6.4	−0.923	0.356	m
		Pre‐/postchange	2.9 ± 1.9	0.9 ± 1.9	−3.405	0.003	m
		Pre‐/postchange *p*	0.003	0.014			w
	Visuomotor constraction immediate memory						
		Pretreatment	13.3 ± 5.7	11.8 ± 6.5	−0.594	0.552	m
		Posttreatment	15.6 ± 5.4	12.3 ± 7.2	−1.056	0.291	m
		Pre‐/postchange	2.4 ± 1.1	0.5 ± 0.9	−2.971	0.001	m
		Pre‐/postchange *p*	0.003	0.102			w
	Visuomotor construction long‐term memory						
		Pretreatment	12.1 ± 5.5	9.3 ± 6.5	−1.885	0.059	m
		Posttreatment	14.1 ± 5.8	9.9 ± 7.4	−2.082	0.037	m
		Pre‐/postchange	2.0 ± 1.3	0.6 ± 1.2	−2.729	0.006	m
		Pre‐/postchange *p*	0.003	0.102			w
	Thinking operations						
		Pretreatment	29.4 ± 2.2	29.2 ± 4.4	−0.730	0.465	m
		Posttreatment	31.7 ± 2.3	29.7 ± 4.6	−1.048	0.284	m
		Pre‐/postchange	2.4 ± 0.8	0.5 ± 0.8	−3.533	0.000	m
		Pre‐/postchange *p*	0.003	0.063			w

*Note:* Values are presented as mean ± standard deviation. Within‐group comparisons were performed using the Wilcoxon signed‐rank test, and between‐group comparisons were conducted using the Mann–Whitney U test. A p value < 0.05 was considered statistically significant.

Posttreatment comparisons of absolute scores demonstrated limited between‐group differences. Analysis of pre‐ to posttreatment change scores revealed significant between‐group differences in orientation, spatial perception, praxis, visuomotor construction, immediate and long‐term visuomotor memory, and thinking operations, with larger changes observed in the experimental group (Table 4).

Within‐group analyses demonstrated significant pre‐ to posttreatment changes across all DOTCA‐Ch subdomains in the experimental group, whereas within‐group changes in the control group were smaller and not consistently significant across domains.

## 4. Dıscussion

In the present study, the effects of a physiotherapy program consisting of SIT combined with balance and coordination exercises were investigated in children with cerebral palsy (CP). The intervention outcomes included balance, functional mobility, functional independence, cognitive performance, and sensory processing characteristics.

The present study evaluated sensory processing patterns using the Sensory Profile. Significant between‐group differences in pre‐ to posttreatment change scores were identified in the Seeking, Sensitivity, and Avoiding subscales, with larger changes observed in the experimental group. Improvements were also observed in several sensory processing domains within the control group; however, these changes were less consistent across subscales. No significant between‐group differences were identified in posttreatment absolute scores. These findings suggest that the intervention was associated with changes in sensory processing patterns, particularly in domains related to sensory seeking, sensitivity, and avoidance.

Baseline demographic characteristics and pretreatment assessments demonstrated general comparability between the experimental and control groups. At the end of the intervention period, improvements were observed in both groups across several outcome measures. Statistically significant within‐group changes were identified in balance, functional independence, functional mobility, and DOTCA‐Ch scores. In addition, analysis of pre‐ to posttreatment change scores demonstrated larger changes in several outcomes in the experimental group receiving individualized SIT; however, these findings should be interpreted cautiously because formal group × time interaction analyses were not performed.

Children with CP commonly present with sensory, cognitive, communication, and motor impairments that may affect functional participation [30].

In selecting participants, we included children aged 6–10 years with GMFCS Levels I and II. Our aim was to examine the contributions of individualized sensory integration (SI) training to cognitive levels and balance in school‐aged children. Although numerous studies exist regarding SI intervention in children aged 0–6 years, research focusing on school‐aged children remains limited. With this study, we aimed to contribute to this gap in the literature.

Children with cerebral palsy frequently exhibit sensory processing difficulties involving vestibular, proprioceptive, tactile, visual, and auditory domains. Sensory processing challenges have been reported in a large majority of children with CP, affecting tactile, proprioceptive, and other sensory modalities and are associated with difficulties in motor planning and functional activities. In addition to sensory issues, children with CP often present with co‐occurring cognitive, perception, and behavioral disturbances that impact participation and daily functioning [31]. Therefore, in planning our treatment program, we incorporated praxis exercises to support participants′ learning processes and cognitive levels in a positive manner.

Franjoine et al. [32] examined the applicability of the PBS in school‐aged children with mild or moderate balance problems and concluded that the scale is a reliable tool for assessing functional balance in this age group. In our study, both groups showed statistically significant increases in Pediatric Berg scores after treatment. The SI group showed an average improvement of 4.1 points, whereas the control group improved by 2.1 points. Although larger changes were observed in the SI group, the between‐group difference did not reach statistical significance. The activity‐ and play‐based structure of SI‐oriented balance tasks involving multisensory input may have contributed to these findings. We attribute this to the fact that, compared with classical balance training, SI‐based balance tasks were performed through activity‐ and play‐based tasks involving multisensory input, possibly providing additional sensory–motor stimulation during balance‐related activities.

Kubilay et al. [33] assessed the effect of balance and posture exercises on functional level in 28 children with mild mental retardation, using the TUG test for functional mobility and the Pediatric Balance Scale for balance assessment. Both groups demonstrated improvements in functional mobility following the intervention, although no significant between‐group difference was observed. Balance significantly affects independence in many daily activities in children with CP. Functional independence in daily activities was assessed using the WeeFIM, which measures the level of dependence in daily activities among preschool and school‐aged children. At baseline, mobility, self‐care, and cognitive subdomains showed impairment, with no significant difference between groups. As expected, posttreatment WeeFIM scores increased significantly in both groups. The SI group showed an average increase of 3.1 points, whereas the control group improved by 1.4 points. Larger improvements were observed in the SI group; however, the absence of formal interaction analyses limits conclusions regarding comparative intervention effectiveness. Cognitive functions such as attention, memory, motor planning, and problem‐solving are important for participation in daily and academic activities in children with CP [34]. The Developmental Test of Cognitive Abilities—Children (DOTCA‐CH) is widely used in clinical practice and research to assess cognitive performance and potential in school‐aged children. The test manual and related validation studies support its use as a reliable assessment of multiple cognitive domains including orientation, memory, visuomotor construction, and thinking operations. Likewise, the adult cognitive assessment derived from the DOTCA framework (DLOTCA) has been employed in clinical research with adult populations, including individuals with stroke, demonstrating acceptable psychometric properties for evaluating cognitive function [28].

Fil et al. [35] examined the effects of sensory integration training in individuals with Parkinson′s disease, comparing a general physiotherapy program with an SI‐enhanced program. Both groups improved on all motor and cognitive measures, but quality of life scores did not change. They concluded that adding SI training to treatment programs yields positive outcomes. In our study, we observed similar improvements in balance, functional independence, DOTCA‐CH outcomes, and sensory profiles.

Although no pretreatment difference was observed between groups, both groups improved after treatment, with statistically significant greater improvement in the SI group. Findings from the DOTCA‐CH assessment indicate that the intervention was associated with improvements across multiple cognitive domains. Greater gains observed in orientation, praxis, visuomotor construction, memory components, and thinking operations suggest a potential enhancement in higher level cognitive organization and problem‐solving abilities. These domains are closely related to functional independence and learning‐related activities, and the observed changes may reflect possible changes in cognitive efficiency rather than isolated skill acquisition. Improvements in cognitive domains assessed by the DOTCA‐CH may support children′s functional participation in learning and daily activities that require planning, memory, and visuospatial processing.

Sensory processing difficulties are well documented in children with cerebral palsy, and research consistently shows that children with CP demonstrate significantly different sensory processing patterns—particularly in tactile, proprioceptive, vestibular, and modulation domains—when compared with typically developing peers. For example, Bar‐Shalita et al. (2009) reported that children with CP show markedly higher sensory modulation difficulties, supporting the clinical use of sensory‐based assessments such as the Sensory Profile in this population [5].

The findings related to behavioral and motor components of sensory processing may indicate changes associated with the intervention beyond general sensory modulation. Greater improvements observed in oral sensitivity, attentional behaviors, perceptual fine motor skills, and emotional responses suggest that the intervention may have contributed to improved adaptive functioning in daily activities requiring integrated sensory‐behavioral responses. In contrast, domains such as endurance and sedentary behaviors appeared less responsive, possibly reflecting more trait‐like characteristics or the need for longer or more targeted intervention strategies. Improvements in behavioral‐ and motor‐related sensory processing domains may facilitate children′s participation in daily routines and self‐regulation during functional activities.

, balance, posture, motor planning, bilateral coordination, and eye–hand coordination. The vestibular system, in particular, plays a major role in postural control. An et al. [9] demonstrated that vestibular rehabilitation positively affects postural control, mobility, emotional well‐being, and social participation in hypotonic CP.

Clark et al. [36] found that 4 weeks of rotational vestibular rehabilitation significantly improved gross motor and postural reflexes in 26 children. Many studies have demonstrated the positive effects of vestibular activities on motor development, balance, and environmental awareness. Similar patterns of improvement were observed in the SI group of our study, possibly related to the emphasis placed on vestibular rehabilitation. Labaf et al. [37] examined the effects of SI therapy on gross motor function in CP and found significant improvement in the SI group after a 12‐week program. Wuang et al. pre‐[38] compared SI therapy, neurodevelopmental treatment, and perceptual–motor approaches in 120 children and reported that SI training resulted in superior improvements in fine motor skills, upper‐extremity coordination, and sensory functions, especially in children with moderate cognitive impairment. Our findings are generally consistent with previous studies reporting beneficial changes following sensory integration‐based interventions.

Reviewing the literature, there are relatively few studies on SI therapy in children with CP, particularly in this age range and using this combination of assessment tools and interventions. Our study therefore provides a novel perspective for physiotherapists working with children with CP.

However, limitations include the small sample size, variety of CP types, study duration, and weekly session frequency. In addition, the use of separate within‐group and between‐group nonparametric analyses, rather than formal repeated‐measures interaction models, limited the ability to directly evaluate group × time interaction effects and may increase the risk of Type I error. Therefore, conclusions regarding comparative intervention effectiveness should be interpreted cautiously. Increasing sample size and therapy frequency may yield more robust results in future studies.

In conclusion, both intervention approaches were associated with improvements in balance, functional mobility, functional independence, cognitive performance, and sensory processing characteristics in children with CP. Larger changes were observed in several outcomes in the group receiving SIT combined with balance and coordination exercises. These findings suggest that incorporating sensory integration–based activities into physiotherapy programs may provide additional support for rehabilitation in children with CP. Integrating SI activities into physiotherapy programs for children with CP may provide additional support for rehabilitation outcomes. However, due to the absence of formal group × time interaction analyses, the comparative effectiveness of the intervention should be interpreted cautiously.

## Author Contributions

**Hande Yılmaz:** conceptualization, investigation, data curation, formal analysis, methodology, resources, writing – original draft. **Feyza Şule Badıllı Hantal:** conceptualization, methodology, supervision, validation, writing – review and editing, project administration.

## Funding

No funding was received for this manuscript.

## Ethics Statement

This study was approved by the *Bahçeşehir University Clinical Research Ethics Committee* (approval date: October 31, 2017).

## Consent

Written informed consent for publication of identifying information/images was obtained from the parents/legal guardians of all participating children.

## Conflicts of Interest

The authors declare no conflicts of interest.

## Data Availability

The data that support the findings of this study are available on request from the corresponding author. The data are not publicly available due to privacy or ethical restrictions.
